# *Babesia ugwidiensis*, a new species of Avian piroplasm from Phalacrocoracidae in South Africa

**DOI:** 10.1051/parasite/2012194375

**Published:** 2012-11-15

**Authors:** M.A. Peirce, N.J. Parsons

**Affiliations:** 1 MP International Consultancy 6 Normandale House, Normandale Bexhill-on-Sea East Sussex TN39 3NZ UK; 2 Corresponding Associate, International Reference Centre for Avian Haematozoa, Queensland Museum PO Box 3300 South Brisbane QLD 4101 Australia; 3 Southern African Foundation for the Conservation of Coastal Birds PO Box 11116 Bloubergrant 7443 South Africa

**Keywords:** *Babesia*, piroplasm, cormorant, marine bird, tick vector, *Babesia*, piroplasme, cormoran, oiseau de mer, tique, vecteur

## Abstract

A new species of haematozoa, *Babesia ugwidiensis* sp. nov. from a cormorant is described. This is the first species of piroplasm to be recorded from the Phalacrocoracidae and the relationship of this parasite to other *Babesia* spp. from marine hosts is discussed.

## Abstract (fr)

Description d’une nouvelle espèce d’hématozoaire, *Babesia ugwidiensis* n. sp., chez des cormorans. Cette espèce est le premier piroplasme décrit des Phalacrocoracidae. La parenté de ce parasite avec les autres espèces de *Babesia* d’hôtes marins est discutée.

## Introduction

New species of haematozoa were recently described from Phalacrocoracidae and Stercorariidae in South Africa (Parsons *et al.*, 2010). As part of the on-going survey of the health of sick and injured marine and coastal birds presented to the Southern African Foundation for the Conservation of Coastal Birds (SANCCOB) for examination and release when possible, new and hitherto undescribed blood parasites have been observed as a result of a range of veterinary investigations to which such birds are subjected, including screening for the presence of blood parasites. A new species of avian piroplasm has been found in several species of cormorants and is herein described. An appraisal of the current status of this and other avian piroplasms described since the last review (Peirce, 2000) is also discussed.

## Materials and Methods

Birds were bled from the medial metatarsal vein as it lies over the tarsal bone with a 23G or 25G needle (depending on the size of the bird). Blood was collected directly from the hub of the needle into a heparinised capillary tube. A thin blood smear was made immediately, air-dried, fixed in methanol for three minutes and stained with modified Wright-Giemsa stain (Kyro-QuickTM stain set, Kyron Laboratories (pty) Ltd, Benrose, South Africa). All slides were initially screened at SANCCOB, each slide being examined for ten minutes, using a 50 × oil immersion lens. A selection of slides found to contain parasites were subsequently re-examined by MA Peirce. These slides were screened under a microscope initially at 160 × and 800 × and then under 1,000 × oil immersion magnification. All images were captured using a digital camera attached to the microscope. Morphometric measurements were obtained using a screw micrometer calibrated against a standard stage micrometer. Due to the small and delicate nature of the parasites, drawings were prepared to provide a clearer morphological picture.

Many of the birds were sampled on a weekly basis while at SANCCOB which allowed the course of infection to be followed in some instances and the parasitaemia monitored. Reference slides have been deposited in the International Reference Centre for Avian Haematozoa (IRCAH) Collection. Any ticks found on birds were carefully removed for subsequent identification.

## Results

Between 2001 and 2011, a total of 851 cormorants were bled at least once. These comprised five species: 689 Cape Cormorant *Phalacrocorax capensis* (56 % positive), 33 Bank Cormorant *P. neglectus* (36 % positive), 57 White-breasted Cormorant *P. carbo* (25 % positive), 64 Crowned Cormorant *P. coronatus* (5 % positive) and eight Reed Cormorant *P. africanus* (13 % positive). The positive birds were all infected with hitherto undescribed intraerythrocytic species of piroplasms, presumably of the genus *Babesia*. The level of parasitaemia in individual birds ranged from 0.1 % to 16.0 %. In some instances the level of parasitaemia rose before falling again, ie the initial parasitaemia in one *P. coronatus* showed a level of 5.0 % on 17 October 2011, which rose to 16.0 % a week later. It was rare to find any ectoparasites other than lice. Only one bird, a *P. capensis* chick, was carrying any ticks on it (12 larvae), which were identified as the argasid *Ornithodoros capensis* (*Carios capensis* is now recognised as *Ornithodoros capensis* (Guglielmone *et al.*, 2010)). Blood smears from this bird when first sampled at three weeks old were negative for parasites, but when sampled again at 4 w.o. (19 December 2011) it was positive for *Babesia* with a parasitaemia of 4.0 %. The bird was re-sampled on 26 December 2011, 03 January 2012 and 09 January 2012 when the parasitaemias were 0.5 %, 6.0 % and 0.5 % respectively. There was no evidence of apparent morbidity due to the infection and the bird was not administered any treatment.

### Taxonomic Review

Family: Phalacrocoracidae (cormorants).

**Fig. 1.**
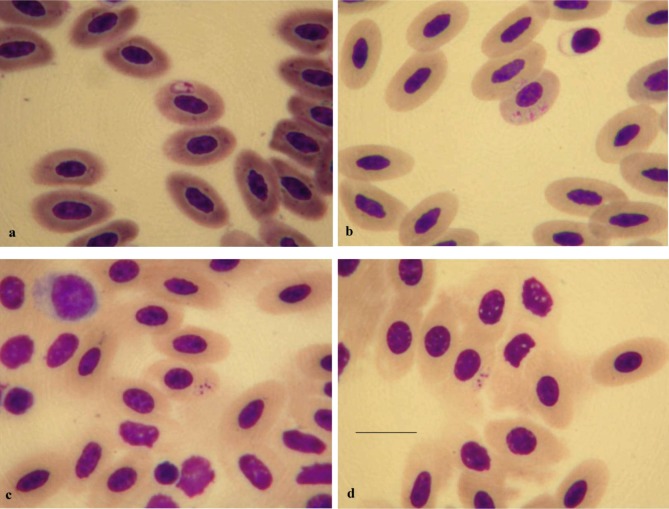
*Babesia ugwidiensis* sp. nov. from *Phalacrocorax carbo*. (a) elongate schizont precursor; (b) three spherical schizont precursors in same cell; (c-d) dividing tetrad schizonts showing proximal position of nucleus within the merozoites. Merozoites are just beginning to separate. Scale bar = 10 μm

**Fig. 2.**
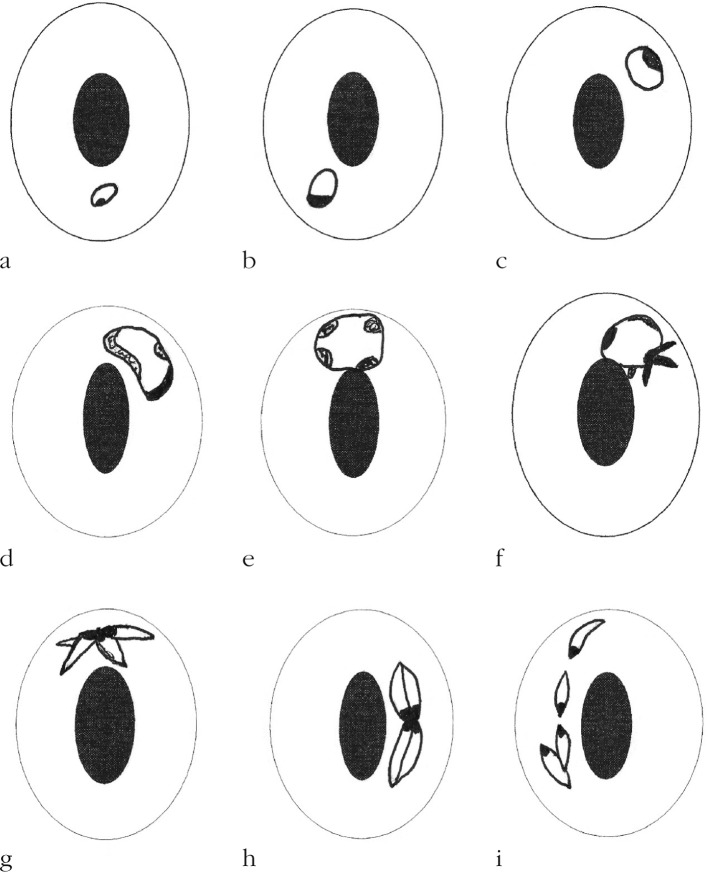
Drawings illustrating the development of *Babesia ugwidiensis* sp. nov. in *Phalacrocorax carbo*. (a) early ring form; (b-c) trophozoites; (d) elongate schizont precursor; (e) spherical schizont precursor showing four chromatin masses; (f) developing amoeboid schizont; (g-h) mature tetrad schizonts; (i) merozoites separated from schizont. Scale bar = 4 μm

Parasite: *Babesia ugwidiensis* sp. nov. (Figs 1a-d, 2 a-i)

Type host: *Phalacrocorax carbo* (L.).

Type locality: SANCCOB, Bloubergrant, South Africa.

Vector: possibly *Ornithodoros capensis* (Neumann, 1901) on circumstantial evidence.

Etymology: named after the Xhosa name for cormorant. Other cormorant species infected with morphologically identical piroplasms: *P. capensis*, *P. coronatus*, *P. neglectus* and *P. africanus*. These presumably occur throughout Africa in other species of Phalacrocoracidae and probably over the geographic range of the family.

### Description

The smallest parasites seen are ring forms comprising a compact mass of chromatin with a pale central cytoplasm measuring 0.5 μm (Fig. 2a). These grow in size (Fig. 2b-c) and gradually show an increase in the chromatin, which begins to divide. These forms either retain their generally round shape (Figs 1b, 2e) and measure 3.11 ± 0.34 μm (range 2.43-3.9 μm) (*n* = 20) or become oval to elongate (Figs. 1a 2d) measuring 4.28 ± 0.46 × 2.5 ± 0.42 μm (range 3.6-5.1 × 1.62- 3.24 μm) (*n* = 13). These schizont precursors which may sometimes appear as amoeboid forms (Fig. 2f) develop into tetrad schizonts (Figs 1c-d, 2g-h) measuring 5.65 ± 0.41 × 2.83 ± 0.26 μm (range 5.04-6.21 × 2.4-3.36 μm) (*n* = 12). The nucleus in the merozoites is proximal and typical cruciform “Maltese-cross” schizont forms are rarely seen. The merozoites, which are elongate and generally pyriform eventually separate (Fig. 2i) and measure 2.62 ± 0.14 × 1.34 ± 0.18 μμm (range 2.22-2.91 × 0.99-1.71 μm) (*n* = 30). After separation the merozoites gradually round up. Where multiple invasion of erythrocytes is seen, this appears to be from the post-schizont merozoites growing within the same host cell, rather than from invasion by other parasites. There is no displacement of the host cell nucleus and no direct evidence of pathogenicity.

### Hapantotype

IRCAH: G 465493 from *P. carbo* coll. Parsons, 5 January 2009, SANCCOB, Bloubergrant, South Africa; deposited in IRCAH Collection, Queensland Museum, Brisbane, Australia.

### Parahapantotypes

IRCAH: G 465494 from *P. carbo* coll. Parsons, 19 January 2009; IRCAH: G 465495 from *P. capensis* coll. Parsons, 5 January 2009; IRCAH: G 465496 from *P. capensis* coll. Parsons, 26 January 2009; IRCAH: G 465497 from *P. capensis* coll. Parsons, 18 July 2011. All from SANCCOB, Bloubergrant, South Africa; deposited in IRCAH Collection, Queensland Museum, Brisbane, Australia.

### Comments

*B. ugwidiensis* sp. nov. is readily distinguished from other *Babesia* spp. of marine birds by the proximal location of the nucleus within the merozoite. It would appear, like most other avian piroplasms, this species is host-specific to the Phalacrocoracidae.

## Discussion

In a review of avian piroplasms of the genus Babesia, Peirce (2000) accepted 13 species as being valid. Since then two additional species have been described, *Babesia kiwiensis* from the kiwi *Apteryx australis mantelli* (Peirce *et al.*, 2003) and *B. uriae* from common murres *Uria aalge* (Yabsley *et al.*, 2009). The description of *B. ugwidiensis* presented here brings the total number of species to 16. *B. ugwidiensis* is a parasite occurring very commonly in *P. carbo* and presumably other cormorants where quite a high prevalence is seen in several species, especially in Cape cormorants. Although some species of avian *Babesia* do cause some degree of pathogenicity and morbidity particularly *B. shortti* in falcons, *B. peircei* in penguins and *B. kiwiensis* in kiwi (Peirce, 2000; Peirce *et al.*, 2003), there is no evidence that *B. ugwidiensis* has any direct observable morbidity in cormorants. However, a high proportion of cormorants received at SANCCOB are emaciated, often due to high worm burdens, and in those cases where *B. ugwidiensis* may also be present as a concomitant infection, it might contribute to the overall morbidity in some birds.

Molecular characterisation of *B. kiwiensis*, *B. poelea* and *B. uriae* confirms that avian piroplasms are more closely related to mammalian *Babesia* than to *Theileria* (Yabsley *et al.*, 2006; Yabsley *et al.*, 2009; Jefferies *et al.*, 2008). It is hoped that an on-going molecular study (Yabsley, *pers. com.*) will confirm also whether *B. poelea* is a valid species or a synonym of *B. peircei* as suggested by Peirce (2000). *B. ugwidiensis* can be easily distinguished morphologically from other *Babesia* spp. from marine birds by the proximal position of the nucleus in merozoites which in *B. peircei*, *B. poelea* and *B. uriae* occur distally. Molecular studies will also have to confirm that the piroplasms found in other cormorant species than *P. carbo* also belong to *B. ugwidiensis*.

At present nothing is known for certain about the vector of *B. ugwidiensis* which until now has been assumed to be a species of ixodid tick, although in colonial ground-nesting species there is always the possibility that argasid ticks of the genus *Ornithodoros* (synonyms: *Alectorobius*; *Carios*) could be involved (Peirce, 2000). The most common species associated with sea birds is *O. capensis*, which Theiler (1959) recorded mainly from nests, although also from penguins and gannets by inference from colonies on the Guano Islands around the Cape. Although not recorded from cormorants in the same area, *O. capensis* has been recorded from the nests of *P. carbo* in Ethiopia and Tanzania (Hoogstraal *et al.*, 1976). While *Ixodes uriae* is the most common ixodid tick collected from sea birds in southern African waters there appear to be no records from cormorants, gannets or penguins (Heyne, *pers. com.*). Since *O. capensis* was the only species of tick to be found on any birds at SANCCOB, from one *P. capensis* chick, which subsequently developed *B. ugwidiensis*, there is a strong possibility that this tick may be the vector, although further study is required to demonstrate this conclusively. Additionally, since the ticks recovered were all larvae, this suggests that trans-ovarial transmission occurs as it does with most species of *Babesia* transmitted by ixodid ticks, as well as trans-stadial. Another factor in favour of *O. capensis* being the vector is provided by the rapid feeding of all stages, larvae, nymphs and adults, all of which feed within an hour or two when conditions are right, due to the anatomy of the mouth parts. Conversely, *I. uriae* takes considerably longer with larvae and nymphs being attached on the host for six-12 days and females about eight days (Frenot *et al.*, 2001). Thus it is surprising that *I. uriae* have not been found on any of the birds examined at SANCCOB. There is ongoing work to identify all species of ectoparasites occurring on the different bird species admitted to SANCCOB.

It is highly probable that ground-nesting marine birds (penguins, gannets and cormorants) which share the same or contiguous nesting areas are sharing the same species of tick, which may be transmitting more than one species of avian *Babesia*. On the available evidence it does appear that argasid ticks may be more important in the transmission of avian *Babesia* spp. in marine birds rather than ixodid ticks.
